# Editorial: Vestibular rehabilitation, neuromodulation and balance in clinical applications of neurology and otoneurology: what is the recent evidence from basic and clinical research?

**DOI:** 10.3389/fresc.2025.1602044

**Published:** 2025-05-08

**Authors:** Michael C. Schubert, Catarina Costa Boffino

**Affiliations:** ^1^ Laboratory of Vestibular NeuroAdaptation, Department of Otolaryngology-Head and Neck Surgery, Johns Hopkins University, Baltimore, MD, United States; ^2^ Department of Health, Physical Therapy Course, Universidade Municipal de São Caetano do Sul (USCS), São Caetano do Sul, Brazil; ^3^ Laboratório de Psicopatologia e Terapêutica Psiquiátrica (LIM 23), Instituto de Psiquiatria do Hospital das Clinicas HCFMUSP, Faculdade de Medicina, Universidade de Sao Paulo, São Paulo, Brazil

**Keywords:** vestibular rehabilitation, BPPV, nystagmus, compensation, neuro-otology, postural control

**Editorial on the Research Topic**
Vestibular rehabilitation, neuromodulation and balance in clinical applications of neurology and otoneurology: what is the recent evidence from basic and clinical research?

Vestibular rehabilitation has its origins in the United Kingdom in the 1940s when patients suffering symptoms of dizziness and imbalance related to either Meineire's disease or traumatic brain injury were tasked to perform a series of visual-vestibular exercises. In recent times, evidence on the clinical utility and mechanisms responsible for vestibular rehabilitation has flourished, including a growing number of investigators engaged in such research. Related topics of interest include anatomical and physiological studies as well as the relevance of the physical and functional impairments related to postural and oculomotor control. The current Research Topic, entitled “Vestibular Rehabilitation, Neuromodulation and Balance in Clinical Applications of Neurology and Otoneurology: What is the Recent Evidence from Basic and Clinical Research?” merges a broad collection of articles captured over a two-year period that represent advanced knowledge and future technologies. Challenging presentations of common clinical diagnoses are included.

Lacour et al. challenge the notion that spontaneous nystagmus is an unmodifiable measure of static compensation, suggesting instead that it can be influenced by vestibular rehabilitation. This reinforces our understanding that the mechanisms of vestibular rehabilitation do indeed engender neuroplasticity and modulation of neuronal networks. The exciting work of Kobel et al. improves computerized dynamic posturography and uses non-linear metrics to identify a pattern-specific sway that distinguishes patients with persistent postural and perceptual dizziness from healthy controls. Wagner and Merfeld further advance posturography by considering medial-lateral sway in addition to anterior-posterior sway and suggest that changes in head position and base of support offer a more challenging task. Harrell et al. reveal that physical therapists do not universally examine for Benign Paroxysmal Positional Vertigo (BPPV) but instead tend to perform clinical testing for BPPV depending on the patient's subjective report. Ludwig and Schubert remind the reader that BPPV can present with atypical nystagmus patterns that warrant critical observation and testing relative to the head position. Meldrum et al. illustrate the novel delivery of vestibular rehabilitation using wearable sensors in persons living with multiple sclerosis to improve their frequency of head motion in addition to reducing the symptoms and impairments related to their dizziness. Xavier et al. used a retrospective design to tackle the complicated, chronic vestibular patient who has not experienced the expected compensation. Their work suggests that vestibular rehabilitation, which includes not only cognitive and emotional tasks but also cervical spine and maxillofacial methods to reduce muscle tension, can further improve rehabilitation outcomes. DiLiberto et al. remind us of the importance of assessing vestibular function in people living with diabetes and provide a rationale for conducting vestibular function tests in this population, in addition to ideas for future research and clinical care. Exciting work from Maruta et al. suggests that attenuating velocity storage by exposing patients with Mal de Debarquement to incremental, low-frequency horizontal rotation coupled with conflicting visual stimuli caused a longer duration of improvement than efforts to correct spatial disorientation without modifying velocity storage. We hope that you will enjoy this special article as much as we have enjoyed curating it.

